# Supplementary material for: Distance-dependent inhibition facilitates focality of gamma oscillations in the dentate gyrus
Source: Nat Commun. 2017 Oct 2;8:758. doi: 10.1038/s41467-017-00936-3 (PMC5624961; doi:10.1038/s41467-017-00936-3)
Supplement: Supplementary file 1 — Supplementary Information [file 41467_2017_936_MOESM1_ESM.pdf]

Distance-dependent inhibition facilitates focality of gamma oscillations in the dentate gyrus

Michael Strüber, Jonas-Frederic Sauer, Peter Jonas and Marlene Bartos

Supplementary information

- Supplementary Figures
- Supplementary Table
- Supplementary References

Supplementary Figures

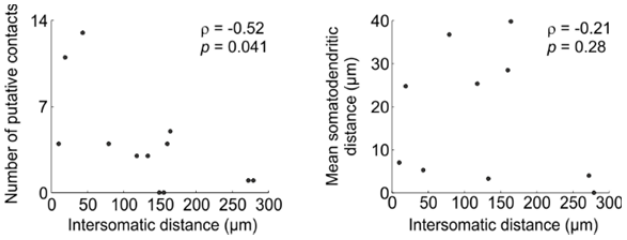

Supplementary Figure 1. Microscopical quantification of synapse number and localization. Individual PIIs (N=3) were filled intracellularly with biocytin in slices of P18 rat dentate gyrus. Biocytin-filled PIIs and remaining PV^+ interneurons were immunofluorescently stained and putative synaptic contacts were identified as close appositions of presynaptic axonal boutons in close vicinity of PV^+ somatodendritic compartments by confocal microscopy. *Left,* the number of putative contacts significantly declined with increasing distance between the pre- and postsynaptic soma. *Right,* the average somatodendritic distance of the identified synaptic contacts remained constant over intersomatic distance. ρ, Spearman's correlation coefficient; *p*, one-tailed analysis.

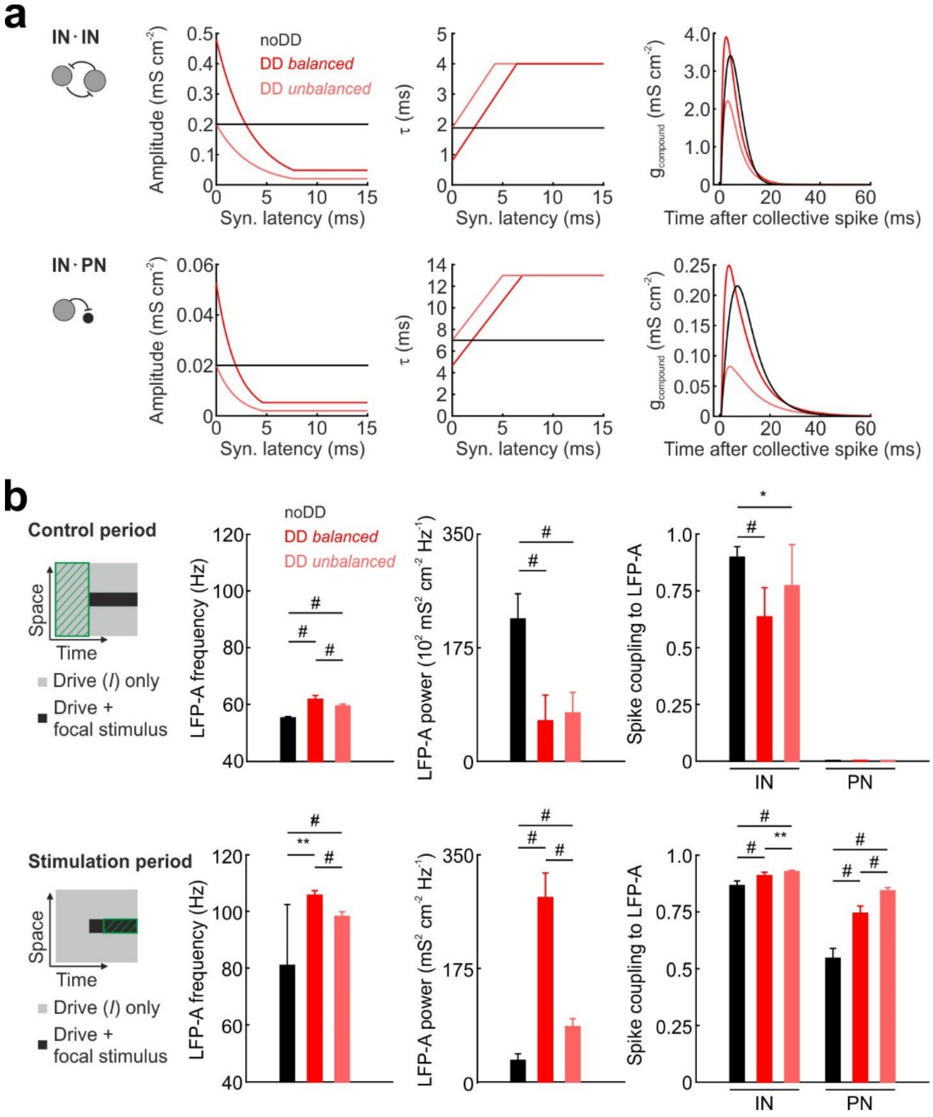

Supplementary Figure 2. Implementation of distance-dependent inhibition in a *balanced* and an *unbalanced* IN-PN network model. (**a**) Distribution of synaptic amplitude (*left*) and τ (*middle*) for IN-IN (*top*) and IN-PN connections (*bottom*) with different synaptic latencies in the noDD (black), *balanced* DD (red) and *unbalanced* DD (light red) network. In the *balanced* DD network, amplitude and τ at closest neighbors were adjusted so that, firstly, the mean τ of all synapses and, secondly, the total compound inhibition were equal to the noDD network. The compound inhibition was defined as the average sum of all synaptic conductance changes in a neuron after synchronous spiking of all presynaptic cells. The resultant theoretical compound inhibition waveforms are shown for all three networks (*right*). Note that the areas under the noDD and *balanced* DD conductance curves are equal. (**b**) Quantification of the oscillatory activity during the control period (t = 0-200 ms) in the whole network (INs #1 to 200, PNs #1 to 800; *top*) and during the stimulation period (t = 200-450 ms) only for the stimulated cells (INs #91 to 110, PNs #361 to 440; *bottom*). The analyzed group of cells and period of time are indicated by the green hatched area in the schematic (*left*). Note that upon focal stimulation, LFP-A power and IN coupling to the LFP-A are higher in both DD networks than in the noDD network. Furthermore, entrainment of PNs at the IN oscillation frequency is less effective in the noDD than in the *balanced* and the *unbalanced* DD network. Bars and error bars represent mean and SD of 10 simulation runs. *, $p < 0.05$; **, $p < 0.01$; #, $p < 0.001$; two-tailed, two-sample t-test.

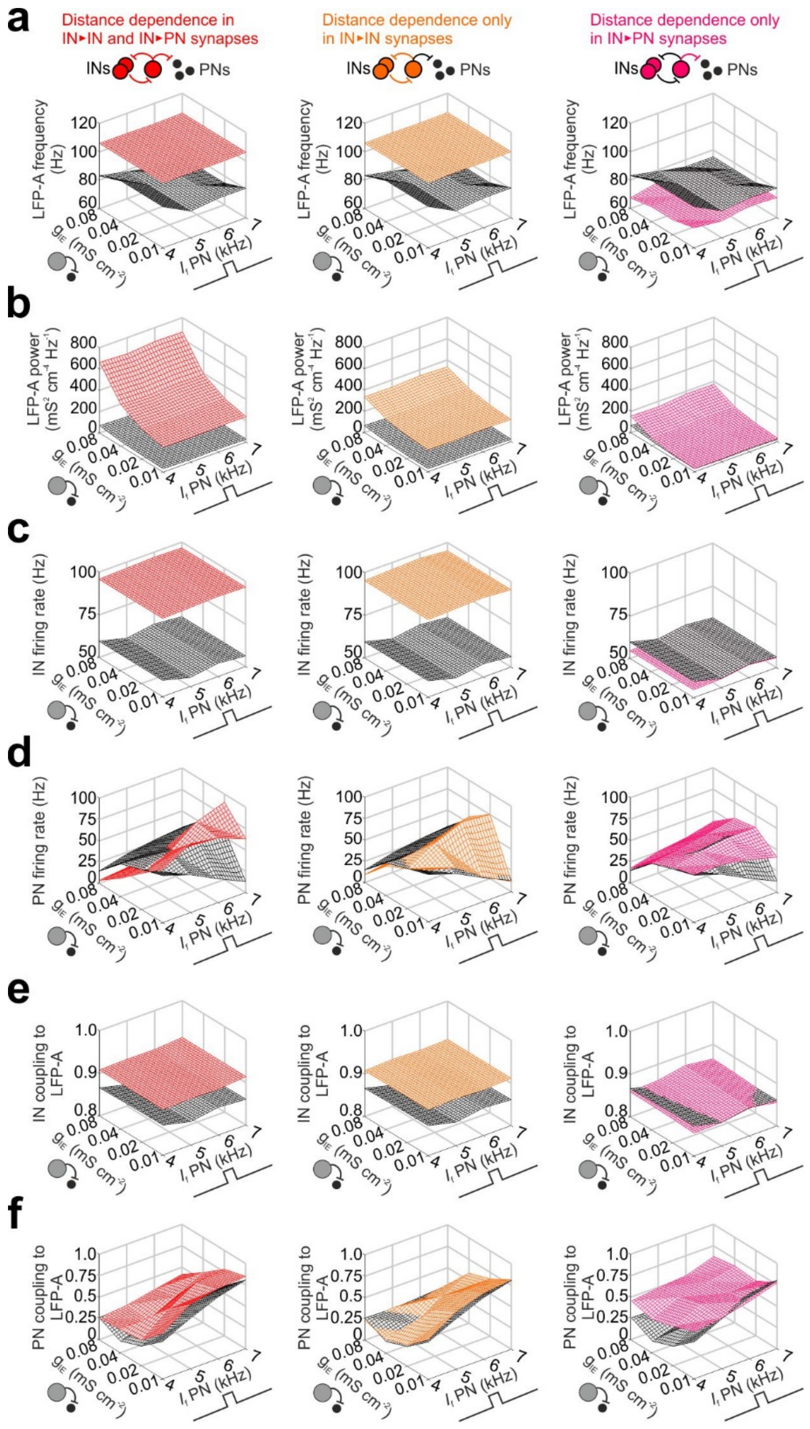

Supplementary Figure 3. Effects of distance-dependent inhibition on focally induced gamma oscillations in IN-PN networks with varying intensities of excitation and inhibition of PNs. Simulations explore the parameter space of the strength of excitation and inhibition of PNs (g_{IE}, peak conductance of IN-PN synapses; I_f PN, average excitatory input frequency of PNs in the center of stimulation). For all combination of parameters, simulations were performed in the noDD model (black surface in all three columns), in the DD model (red surface in the left column) and in network models with distance-dependent inhibition only in IN-IN synapses (DD II, orange surface, middle column) or in IN-PN synapses (DD IE, purple surface, right column). Oscillation properties such as the dominant LFP-A oscillation frequency (**a**), corresponding LFP-A power (**b**), average firing rates of INs (**c**) and PNs (**d**) and strength of coupling of IN and PN spikes to the LFP-A (**e** and **f**, respectively) were analyzed for the stimulated cells (INs #91 to 110 and PNs #361 to 440). Introducing distance-dependence to IN-IN and IN-PN connection can each improve PN spike coupling to the ongoing LFP-A (f). All values are averages of 10 simulation runs.

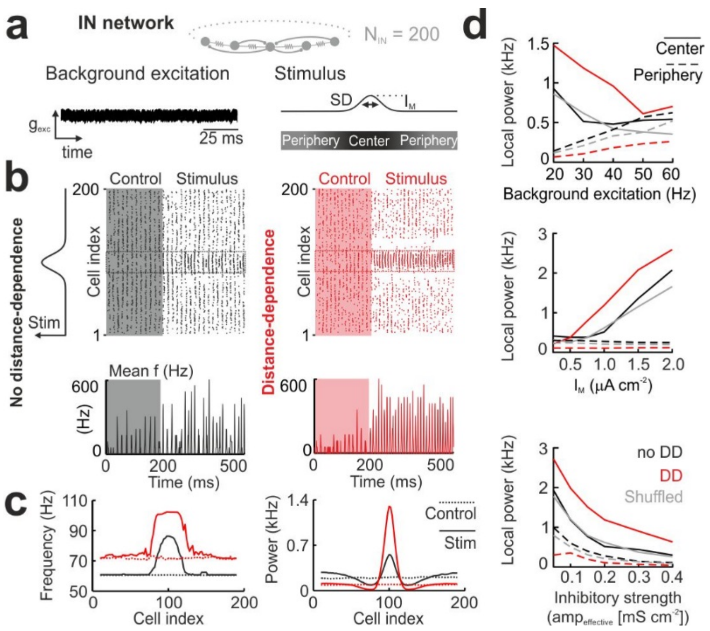

Supplementary Figure 4. The ratio between background excitation and stimulus intensity determines focal gamma power in an IN network model. (**a**) Schematic illustrates the design of network simulations. *Top*, the IN network model consisted of 200 circularly arranged INs interconnected by chemical and electrical synapses. *Bottom left*, all cells received throughout control and stimulation period a Poisson-distributed train of excitatory synaptic inputs. *Bottom right*, a central region of the network was additionally stimulated. The stimulus was Gaussian shaped with the SD of 10 cells and a maximal strength of I_M at the center of stimulation. The network periphery remained unstimulated (see Materials and methods). (**b**) *Top*, raster plots of IN activity in the noDD network (black) and the DD network (red). Gray and red shadows highlight the control period without focal stimulation. *Bottom*, mean momentary firing frequency of INs in the center indicated by boxes in the corresponding raster plots. (**c**) Mean oscillatory frequency (*left*) and firing rate power (*right*) of local groups of INs before (dashed lines) and during focal stimulation (continuous lines). (**d**) Power of IN activity in the center (continuous lines) of stimulation and the periphery (dashed lines) for different background activities in the network (background excitation, *top*), focal stimulation intensity I_M (*middle*) and inhibitory strength (amp$_{effective}$, *bottom*). The power in the center of focal oscillations depends on two parameters: The strength of focal excitation (*top*) and excitatory strength in the surrounding network (*middle*). The higher the difference between central and peripheral stimulation, the more powerful is the focal gamma activity. Note that for all conditions tested, gamma power is always higher in the DD than in either the noDD or the shuffled network, indicating the strong robustness of the finding and its independence of the precise setting of network parameters. Black, noDD; red, DD network; gray, shuffled network with randomly shuffled synaptic properties of the DD network. Values in (c) and (d) are averages of 50 simulation runs.

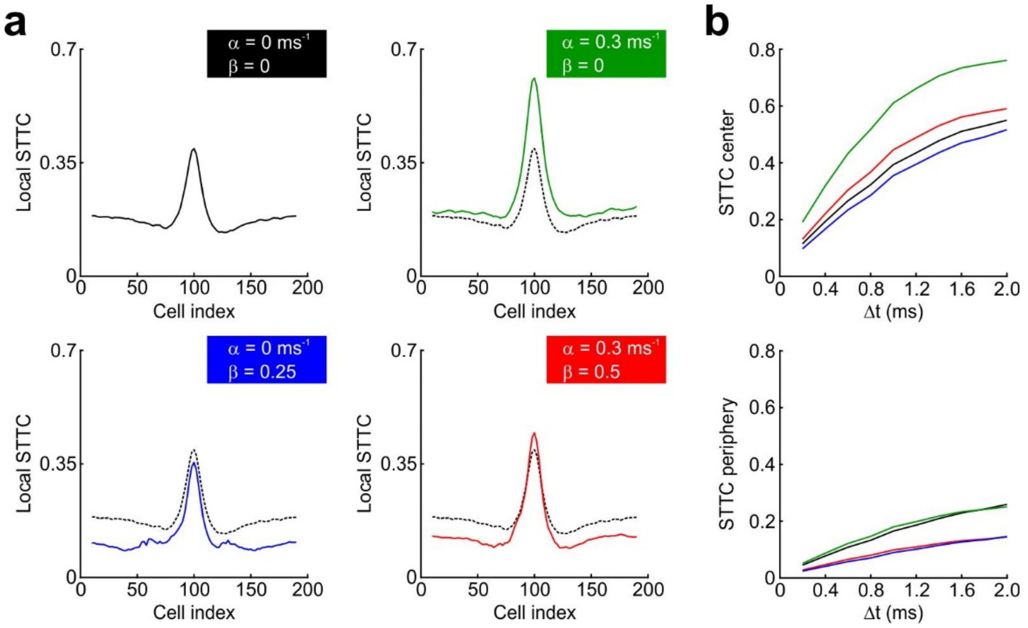

Supplementary Figure 5. Measuring increased synchrony in central DD IN networks using the spike time tiling coefficient (STTC). In order to rule out direct effects of higher recruitment in DD networks on coherence of gamma oscillations (cf. **Fig. 5a**), synchrony of IN firing was quantified by the STTC, a measure for spike train correlations independent of firing rates described in Cutts and Eglen (2014)[1]. (**a**) STTC was quantified in a window of 20 INs, which was slid over the complete network. As shown for the coherence (κ), STTC quantification resulted in an elevated synchrony in the center of the DD network and reduced synchrony in the periphery of the stimulation. (**b**) Effects of distance-dependent inhibition in synchrony of central and peripheral INs was independent of Δt, the only free variable in the definition of STTC. All values are averages of 50 simulation runs.

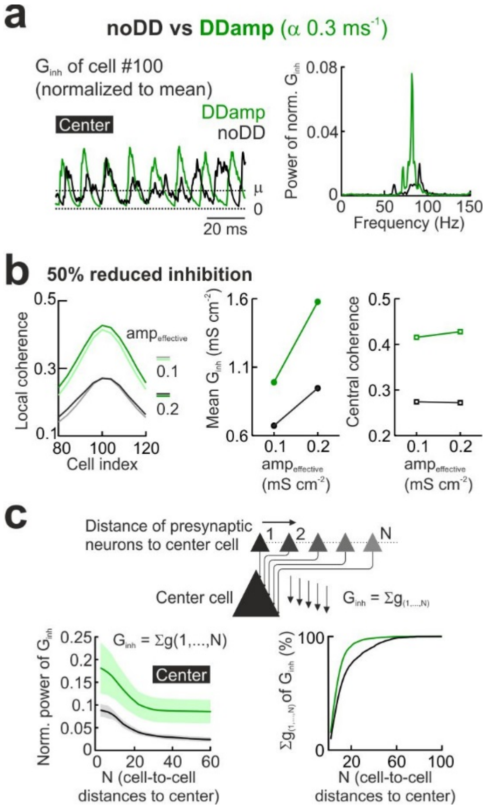

Supplementary Figure 6. Circuit mechanisms underlying focal synchronization by distance-dependent amplitudes in IN networks. (**a**) *Left*, superposition of the total inhibitory conductance (G_{inh}) normalized to its mean measured in a central cell (#100) of the noDD network (black) and a network with only distance-dependent amplitudes (DDamp; α = 0.3 ms^{-1}, green) during focal stimulation. *Right*, the power analysis of normalized G_{inh} in central cells (#99-101) of both networks shows that the periodicity of the inhibitory signal is markedly higher in the DDamp network. (**b**) Effect of the reduction of the inhibitory conductance by 50% ($amp_{effective}$ changed from 0.2 to 0.1 mS cm^{-2}) on focal synchronization to distinguish between the influence of strength and periodic shape of G_{inh} on the observed improvement of central coherence. *Left,* expanded view of the central coherence peaks. *Middle*, reducing $amp_{effective}$ by 50% indeed strongly reduces the average inhibitory conductance per cell in the center. *Right*, reducing $amp_{effective}$ does not influence central coherence. (**c**) *Top*, schematic illustrates unitary synaptic inputs converging from presynaptic neurons with different connection distances onto a postsynaptic central cell, thereby producing G_{inh}. *Bottom left*, power of normalized total G_{inh} recorded from a center cell during central stimulation for 1000 ms is plotted against the number of presynaptic cells, which was systematically increased by including more and more remote cells. Normalized power monotonically decreases as increasing numbers of remote cells are included, indicating that remote cells interfere with periodicity of G_{inh} in the center. Lines with shaded areas indicate mean ± SD. *Bottom right*, cumulative increase in the integral of G_{inh} in a central cell in dependence on the distance of presynaptic interneurons. The contribution of inputs from close neighbors to the total G_{inh} is larger in the DDamp than in the noDD network. Values in (b) and (c) were obtained from 5 simulation runs.

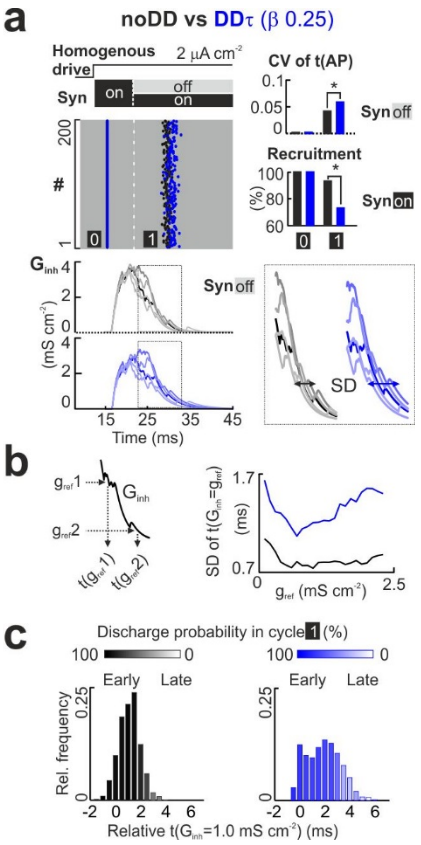

Supplementary Figure 7. Circuit mechanisms underlying the desynchronizing effect of distance-dependent time courses in IN networks. (**a**) *Top*, distance-dependence in τ reduces precision in action potential timing (t(AP)) and recruitment of INs in a network with only distance-dependent τ (DDτ; β = 0.25, blue) in comparison to the noDD (black) network. *Left*, both networks received a homogeneous tonic excitatory drive (I = 2 μA cm^{-2}; noise = 0%). Raster plot shows that all 200 cells (#) discharged at identical time points in cycle ‘0’. Under this condition, only compound G_{inh} determines t(AP) in the next cycle '1'. *Right*, bar graphs summarize coefficient of variation (CV) of t(AP) and the relative recruitment of cells during cycle ‘0’ and ‘1’ of both networks (*, $p < 0.001$, two-sample t-test). *Bottom left*, superposition of the compound G_{inh} depicted from 5 cells in the network (cycle '0'); synapses were switched off before a second action potential was generated. Boxes are shown at higher magnification on the right. Note that the jitter of the decay phase of G_{inh} was higher (double arrow) in networks with distance-dependence in τ. (**b**) *Left*, magnification of the decay of G_{inh}. Two time points ($tg_{ref}1$, $tg_{ref}2$) are highlighted (arrow) at which G_{inh} crossed during its decay a defined conductance value (g_{ref}). *Right*, SD of the g_{ref}-crossing times ($t(g_{inh}=g_{ref})$) in 100 cells is plotted for various values of g_{ref}. SD of g_{ref}-crossings is significantly higher in the $DD^{0|0.25}$ network ($p < 0.01$, two-sample t-test). (**c**) Histograms of the relative g_{ref}-crossing times (g_{ref} = 1.0 mS cm^{-2}). Bar color indicates the probability for a given cell to discharge an action potential during cycle ‘1’. Crossing times were defined relative to the time point when the first-firing cell in cycle ‘1’ crossed g_{ref}. Note that g_{ref}-crossing times occur in the $DD^{0|0.25}$ network later than in the noDD network thereby resulting in an increased silencing of INs. *, $p < 0.001$. All values were obtained from 5 simulation runs.

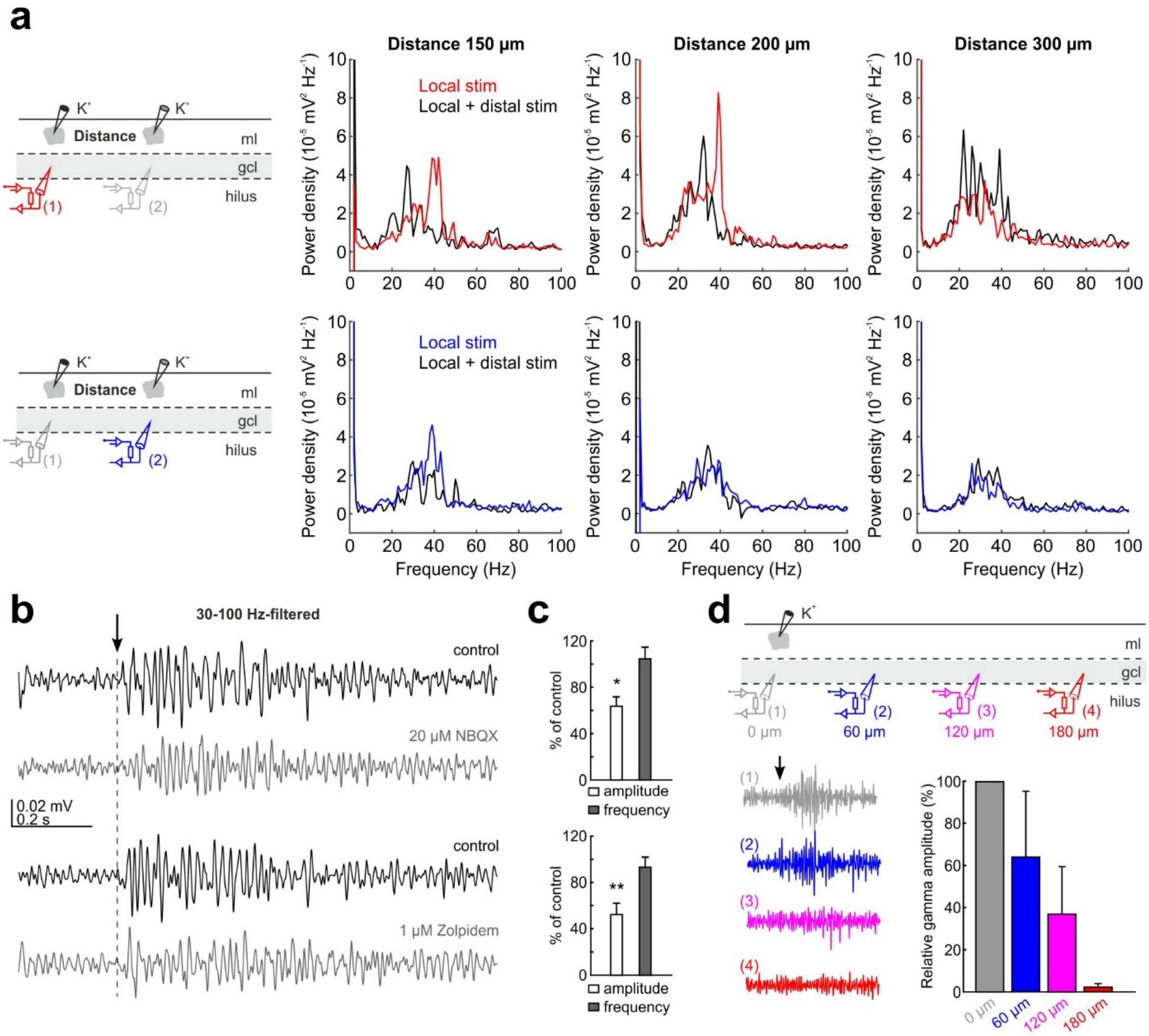

Supplementary Figure 8. Characteristics of focally induced gamma oscillation in dentate gyrus slices. (**a**) *Left*, experimental setup with two KCH_3SO_4 filled puff pipettes in the molecular layer (ml) of rat dentate gyrus slices in variable distances from each other. Two extracellular recording electrodes were positioned at the according local positions in the granule cell layer (gcl). *Right*, average power spectra (N = 3 stimulations, 1 slice) recorded at site 1 (*top*) or site 2 (*bottom*) for different inter-stimulation distances. Colored lines indicate oscillations evoked after local stimulation only. Black lines show power spectra recorded upon simultaneous local and distal stimulation. (**b**) 30-100 Hz filtered LFP traces recorded in the gcl after local puff application of KCH_3SO_4 in the ml. *Top*, puff-evoked gamma activity before and after bath-application of 20 µM NBQX. *Bottom*, filtered LFP traces before and after application of 1 µM zolpidem. Arrow and vertical dashed line indicate the beginning of the puff application. (**c**) Quantification of the experiments shown in (b). Gamma amplitude but not dominant oscillation frequency is reduced by the AMPA/kainate receptor blocker NBQX (N = 6 experiments). Similarly, the $GABA_A$ receptor modulator zolpidem reduces amplitude but not frequency of evoked gamma oscillations (N = 8 experiments). Consistent with an ING mechanism[2], inhibiting AMPA receptor mediated excitation reduces power of oscillations but does not completely inhibit them. In contrast, bath application of SR95531, a specific $GABA_A$ receptor blocker, almost completely abolishes any oscillatory activity in the gamma range (25-45 Hz power integral: control, 4.86×10^{-4} mV^2 Hz^{-1} versus SR95531, 1.12×10^{-4} mV^2 Hz^{-1}; N = 1 experiment, 3 stimulations)[2]. (**d**) Top, schematic of the experimental setup analyzing the spatial spread of focally evoked gamma bursts by recording the LFP in the gcl at different distances from the stimulation pipette located in the ml. Bottom left, representative 30-100 Hz filtered LFP recordings upon stimulation at 0 µm in different distances. Bottom right, normalized gamma amplitude is substantially reduced at distances ≥180 µm (N = 3 experiments). *, $p < 0.05$; **, $p < 0.01$; paired t-test.

Supplementary Table

	IN-PN network	IN network
**Topology**		
N(IN)	200	200
N(PN)	800	
Convergence (IN-IN)	60	60
Convergence (IN-PN)	20	
Convergence (PN-IN)	50*	
SD (IN-IN)	25	25
SD (IN-PN)	25	
SD (PN-IN)	10*	
Synaptic delay (variable part)	0.2 ms per IN-IN distance	0.2 ms per IN-IN distance
Synaptic delay (constant part)	0.5 ms	0.5 ms
Electrical coupling (IN-IN)	4 of 8 nearest neighbors	4 of 8 nearest neighbors
**Cell models**		
IN conductances	passive leak, g(Na_v), g(K_{dr}) (a)	passive leak, g(Na_v), g(K_{dr}) (a)
PN coductances	passive leak, g(Nav), g(Kdr), g(K_A), g(K_M) (b)	
V_{rest} (IN)	-65 mV	-65 mV
V_{rest} (PN)	-75 mV	
R_m (IN)	7.6 kΩ cm^{-2}	7.6 kΩ cm^{-2}
R_m (PN)	38.0 kΩ cm^{-2}	
C_m (IN)	0.93 µF cm^{-2}	0.93 µF cm^{-2}
C_m (PN)	1.01 µF cm^{-2}	
Surface area (IN)	100 μm^2	100 μm^2
Surface area (PN)	100 μm^2	
**Distance-dependence**		
α (IN-IN)	0.3 ms^{-1}	0.3 ms^{-1}
α (IN-PN)	0.5 ms^{-1}	
β (IN-IN)	0.5	0.5
β (IN-PN)	1.2	

(a) Wang and Buszaki (1996)[3]; (b) Hemond et al. (2008)[4]

Continued next page.

	IN-PN network	IN network
**Synapses**		
$amp_{effective}$ (IN-IN)	0.2 mS cm^{-2}	0.2 mS cm^{-2}
$amp_{effective}$ (IN-PN)	0.1 mS cm^{-2}	
amp (PN-IN)	0.05 mS cm^{-2} *	
τ_{rise} (IN-IN)	0.16 ms	0.16 ms
τ_{rise} (IN-PN)	0.16 ms	
τ_{rise} (PN-IN)	0.2 ms*	
Mean τ_{decay} (IN-IN)	1.88 ms	1.88 ms
Mean τ_{decay} (IN-PN)	7.0 ms	
τ_{decay} (PN-IN)	1.0 ms *	
E_{syn} (IN-IN)	-55 mV	-55 mV
E_{syn} (IN-PN)	-65 mV	
E_{syn} (PN-IN)	0 mV *	
g_{gaps} (IN-IN)	0.01 mS cm^{-2}	0.01 mS cm^{-2}
**Excitatory Drive**		
mean f(IN)	300 Hz	300 Hz
mean f(PN)	500 Hz	
amp(IN)	0.01 mS cm^{-2}	0.01 mS cm^{-2}
amp(PN)	0.02 mS cm^{-2}	
τ_{rise} (IN)	0.1 ms	0.01 ms
τ_{rise} (PN)	0.1 ms	
τ_{decay} (IN)	2 ms	2 ms
τ_{decay} (PN)	2 ms	
**Stimulus**		
Format	Square, synaptic train	Gaussian, current injection
Strength at center (IN)	600 Hz	1 μA cm^{-2}
Strength at center (PN)	6000 Hz	
Spatial dimension	width = 20 IN indices	SD = 10 IN indices

Supplementary Table 1. Summary of network model parameters. *, parameters only valid for simulations including PN-IN feedback synapses (**Fig. 4**).

Supplementary References

1. Cutts, C. S. &Eglen, S. J. Detecting pairwise correlations in spike trains: an objective comparison of methods and application to the study of retinal waves. *J. Neurosci.* **34,** 14288–14303 (2014).
2. Towers, S. K. *et al.* Fast network oscillations in the rat dentate gyrus in vitro. *J. Neurophysiol.* **87,** 1165–1168 (2002).
3. Wang, X. J. &Buzsáki, G. Gamma oscillation by synaptic inhibition in a hippocampal interneuronal network model. *J. Neurosci.* **16,** 6402–6413 (1996).
4. Hemond, P. *et al.* Distinct classes of pyramidal cells exhibit mutually exclusive firing patterns in hippocampal area CA3b. *Hippocampus* **18,** 411–424 (2008).
